# Primary care referral to multidisciplinary high risk foot services – too few, too late

**DOI:** 10.1186/s13047-015-0120-7

**Published:** 2015-11-14

**Authors:** D Plusch, S Penkala, HG Dickson, M Malone

**Affiliations:** Western Sydney University, Campbelltown Campus, Campbelltown, Sydney, NSW 2560 Australia; Ambulatory Care, Liverpool Hospital, Locked Bag 7103, Liverpool, NSW 2170 Australia; Department of Podiatric Medicine, High Risk Foot Service, Liverpool Hospital, Locked Bag 7103, Liverpool, NSW 2170 Australia; LIVE DIAB CRU, Ingham Institute of Applied Medical Research, Liverpool, NSW 2170 Australia

## Abstract

**Background:**

To determine if patients with no contact with a multi-disciplinary team High Risk Foot Service (MDT-HRFS) had an increased rate of hospital admission for diabetes foot infection compared to patients with contact. Secondary aims were to report on clinical outcomes.

**Methods:**

A retrospective study was conducted at a major tertiary referral hospital in metropolitan Sydney over 12 months. An ICD-10 search of the electronic medical record system and paper medical charts identified patients with diabetes mellitus (type 1 or type 2) and a primary admission for diabetes foot infection (DFI). Patients were categorised as having contact or no contact with an MDT-HRFS.

**Results:**

One hundred ninety-six hospital admissions (156 patients) were identified with DFI over a 12-month period. Patients with no contact with a MDT-HRFS represented three quarters of admissions (no contact = 116, 74.7 % vs. contact = 40, 25.6 %, *p* = 0.0001) and presented with more severe infection (no contact = 39 vs. contact = 12). The odds of lower extremity amputation increased five-fold when both no contact and severe infection occurred in combination (no contact with severe infection and amputation = 34, 82.9 % vs. contact with severe infection and amputation = 7, 17.1 %, OR 4.9, 95 % CI 1.1–21.4, *p* = 0.037). Using estimates of both the contact and no contact population of people with diabetes and high-risk feet and assuming the risk of amputation was the same, than the number of expected amputations in the no contact group should have been 55, however the observed number was 77, 22 more than expected (*p* = 0.0001).

**Conclusions:**

Patients with no contact with a MDT-HRFS represented the majority of admissions for DFIs to a tertiary referral hospital. This group on population estimates appears to be at high risk of amputation of the lower extremity and therefore early referral of this high-risk group might lower this risk.

## Background

Foot infections are one of the most common causes of hospitalisation in people with diabetes [1, 2] with up to 85 % of cases proceeding to a diabetes related lower extremity amputation [3]. Most frequently DFIs are preceded by ulceration, where a break in the protective barrier of the skin leaves a portal of entry for invading pathogenic organisms. The deficit in the immune-mediated response in people with diabetes may increase the risk and severity of foot infections but the exact underlying process responsible for this deficit, remains poorly characterized [4]. Early recognition and appropriate management of diabetes related foot pathology preceding DFI’s therefore is essential.

Primary care practitioners while providing the majority of medical care for people with diabetes, also play an important role in routine screening, identifying the risk of diabetes related foot pathology and referral needs [5]. High risk foot stratification includes two or more risk factors (peripheral neuropathy, peripheral vascular disease, deformity) and/or a history of ulceration, and/or amputation [6]. Clinical guidelines recommend primary referral of people with a high risk foot to a MDT-HRFS with specialist care from medical, surgical, nursing, podiatry and allied health professionals [5, 7, 8].

Emergency referral to a MDT-HRFS within 24 h is recommended when there is a new ulcer, swelling or foot discolouration [8]. Grading severe infection on the Infectious Disease Society of America (ISDA) guidelines, predicted hospitalisation and amputation rates 89 and 70 % respectively [6]. High-risk foot services have demonstrated reductions in hospital admissions [9], amputations [10–16] and length of stay (LOS) [9, 12, 13, 16–18] for patients known to the service. However, despite clinical success rates and guidelines, many people with diabetes do not receive routine screening for diabetes related foot pathology to enable appropriate early referrals [5].

While the main focus within the literature surrounds MDT-HRFS and the reduction of DRLEA rates, there has been no comparison of hospital admissions for DFIs between people with contact and no contact to a MDT-HRFS. The primary aim of this study was to determine if patients with no contact to a MDT-HRFS were associated with increased numbers of hospital admission for diabetes foot infection. Secondary aims were to report on clinical outcomes in patients hospitalised for DFI, with a focus on any surgical procedures required, LOS, infection classification and cost.

## Methods

A retrospective study was conducted at a major tertiary referral hospital in metropolitan Sydney from 1^st^ January 2012 to 1^st^ January 2013. Ethical approval was obtained from South Western Sydney Local Health District Research and Ethics Committee. Patient information including age, gender, medical history, clinical, laboratory, operative report data and hospital metrics were collected Patients were eligible if they had either type 1 or type 2 diabetes with a primary admission for DFI. Patients were identified using the ICD-10 coding system.

District-linked electronic medical records and paper medical charts were cross-referenced and used to identify patients with ‘contact’ and ‘no contact’ to the MDT-HRFS. Patients with contact were defined as any registered patient attending an outpatient appointment with the MDT-HRFS in the preceding 12 months prior to admission. A MDT-HRFS was defined according to Agency for Clinical Innovation, Endocrine Network standards for high-risk foot services in New South Wales, Australia. There are four MDT-HRFS in the administrative district, each located in a public hospital outpatient area.

People with diabetes and peripheral neuropathy constitute a group at high risk for lower limb amputation as a consequence of ulceration and infection. While neuropathy has been identified as an etiology in the majority of DFI foot ulcerations [2, 19], peripheral vascular disease is present in over half [2] of those requiring hospitalisation and is a predictor of poor healing [2]. The size of this group in our Health District is not known, but estimates can be generated. The population of the Health District is 820,000. Approximately 8 % will have diabetes [19], and of these about 60-70 % will have peripheral neuropathy and or peripheral arterial disease. The severity of the neuropathy and or peripheral arterial disease is likely to be normally distributed, and 20 % could be categorized as being in the high risk group [20]. This gives a target population at high risk of diabetic foot disease of approximately 8500 people. There are approximately 2500 ‘known’ patients on the patient register of the High Risk Foot Clinic at our hospital location. ‘Unknown’ patients to the High Risk Foot Clinic with high risk foot pathology are estimated to be around 6000, derived from subtracting the ‘known’ patients from the target at risk population of 8500.

Outcomes of interest were: any foot surgery or vascular procedure associated with the index admission, the length of hospital stay, severity of infection (using current Infectious Disease Society of America guidelines for diabetes foot infection [2], inflammatory markers, culture results, and estimated hospital costs. Past medical history including comorbid variables in addition to clinical and laboratory data were confirmed using current diagnostic guidelines and obtained from both electronic medical records and paper charts. In particular, PAD was confirmed via documented clinical assessment and or in combination with available vascular studies. Diabetes peripheral neuropathy were confirmed through available clinical indicators including a modified neuropathic disability score >6 [21] absent 10 g monofilament in >3 places on the foot [22] and or available diagnostic electrophysiological studies.

Identified patients for the study were split into two cohorts, contact and no contact to a MDT-HRFS.

Costs for each inpatient separation were calculated utilizing AR-DRG (Independent Hospital Pricing Authority). All values are represented in AUS $. For each patient, the admission, discharge date, principal diagnosis, principal procedure and co-morbidities were extracted and cross checked between electronic records and paper charts.

The cost of each individual patient episode of admission was calculated. Multiple procedures during a single admission were not calculated, and the cost was attributed to the highest surgical procedure cost. Thus if a patient required revascularization and amputation, the cost for amputation would be calculated as it is the higher costing procedure. The cost of multiple admissions within the 12-month period was also calculated for individual patients.

### Statistical analysis

Statistical analysis was undertaken using IBM Statistical Packages for the Social Sciences (SPSS) Version 21.0 for Windows (SPSS Inc, Chicago, IL, USA). Unpaired t tests and chi square with risk ratio were employed in testing differences between cohorts. Mann-Whitney *U* test was used for nonparametric data. A histogram for hospital metric data was performed to look for outliers and normality. For all comparisons and modelling, the level of significance was set at *p* <0.05.

## Results

A total of 196 hospital admissions (156 patients) were identified from the ICD-10 search over the 12 month study period. Of the 156 patients, the majority had no contact with a MDT-HRFS (no contact = 116, 74.7 % vs. contact = 40 patients, 25.6 %, *p* = 0.0001). Patient characteristics including age, gender, medical history, clinical, laboratory and surgical procedures are identified in Table 1.

**Table 1:** Patient characteristics for contact or no contact with a MDT-HRFS

	Contact (*n* = 40)	No Contact (*n* = 116)	Total (*n* = 156)	*p*-value
Demographics				
Mean Age (±SD)	63.4 (±11.8)	65.5 (±14.1)	65 (±13.5)	0.40
Male, n (%)	27 (67.5)	78 (67.2)	105 (67.3)	
Female, n (%)	13 (32.5)	38 (32.8)	51 (32.7)	0.98
Medical history				
Diabetes Type 1, n (%)	4 (10.0)	8 (6.9)	12 (7.7)	
Diabetes Type 2, n (%)	36 (90.0)	108 (93.1)	144 (92.3)	0.54
Duration of Diabetes (±SD)	15.5 (±5.7)	15.8 (±8.5)	15.7 (±7.8)	0.86
HbA1C (mmol/mol)/IFCC	8.4 (±2.2)/68	8.7 (±2.6)/72	8.6 (±2.5)/70.5	0.44
Peripheral Neuropathy, n (%)	35 (87.5)	97 (83.6)	132 (84.6)	0.56
Peripheral Arterial Disease, n (%)	33 (82.5)	95 (81.9)	128 (82.1)	0.93
Ischemic Heart Disease, n (%)	20 (50)	58 (50)	78 (50)	1.00
Hypertension, n (%)	36 (90)	98 (84.5)	134 (85.9)	0.39
CKD stage 5, n (%)	9 (22.5)	19 (16.4)	28 (17.9)	0.21
eGFR (±SD)	41.3 (±26.5)	47.8 (±28.7)	46.2 (±28.2)	0.001*
Length of Stay, n (IQR)	8 (7–12)	11 (6–24)	10 (10–19.5)	0.63
LAB				
White Cell Count, n (IQR)	10.75 (8–15)	12.1 (10–15)	11.8 (9–15)	0.24
Presenting ESR (mmol/L), n (IQR)	57 (34–86)	57 (26–77)	57 (27–77)	0.69
Presenting CRP (mg/l), n (IQR)	76.35 (23–155)	69.75 (32–172)	73.7 (29–166)	0.99
Infection severity				
IDSA grade Moderate, n (%)	28 (70 %)	77 (66.4 %)	105 (67.3 %)	0.68
IDSA grade Severe, n (%)	12 (30 %)	39 (33.6 %)	51 (32.7 %)	0.68
Texas classification				
1B, n (%)	1 (2.5)	6 (5.2)	7 (4.5)	0.48
1D, n (%)	6 (15)	21 (18.1)	27 (17.3)	0.66
2B, n (%)	3 (7.5)	11 (9.5)	14 (9)	0.71
2D, n (%)	12 (30)	52 (44.8)	64 (41)	0.10
3B, n (%)	3 (7.5)	5 (4.3)	8 (5.1)	0.43
3D, n (%)	15 (37.5)	21 (18.1)	36 (23.1)	0.01*
Surgery type				
Surgical debridement, n (%)	7 (17.5)	28 (24.1)	35 (22.4)	0.39
PTA, n (%)	6 (15)	34 (29.3)	40 (25.6)	0.08*
Bypass, n (%)	0 (0)	3 (2.6)	3 (1.9)	0.31
Amputation, n (%)	23 (57.5)	77 (66.4)	100 (64.1)	0.32
Forefoot/digital, n (%)	16 (40)	51 (44)	67 (42.9)	0.66
TMA, n (%)	0 (0)	0 (0)	0 (0)	
BKA, n (%)	8 (20)	18 (15.5)	26 (16.7)	0.51
AKA, n (%)	0 (0)	8 (6.9)	8 (5.1)	0.09*

*ESR* erythrocyte sedimentation rate, *CRP* c-reactive protein, *CKD* chronic kidney disease, *eGFR* estimated glomelular filtration rate, *PTA* percutaneous transluminal angioplasty

*p-value <0.05

### Surgery

A large number of patients admitted for DFI required a surgical procedure (*n* = 126 out of 156, 81 %) and the majority of these were undertaken in patients with no contact (*n* = 94 out of 126, 74.7 %). Amputation was the most common surgical intervention (*n* = 100 out of 126, 72 %) with lower extremity amputation being undertaken more frequently in patients with no contact (no contact = 77, 82 % vs. contact = 23, 72 %, *p* = 0.23). Population estimates for our groups suggested that admission and amputation in the no contact group were over represented, the expected number of amputations should have been 55, however the observed number was 77, 22 more than expected (*p* = 0.0001).

Revascularisations utilising percutaneous transluminal angioplasty (PTA) were performed in 34 (36 %) of no contact patients and 6 contact (19 %) patients with a trend towards significance (*p* = 0.054). Regardless of contact status, digital amputations were the most commonly performed amputation (*n* = 67, 43 %)

### Length of stay

On average, the median LOS in the no contact group was 3 days longer than those with contact (contact = 8 days, IQR 7 to 12 vs. no contact = 11 days, IQR 6 to 24, *p* = 0.063). LOS was influenced by the requirement to undergo a surgical procedure. Regardless of contact status, the LOS for admissions not requiring surgery was 7 days (IQR 5 to 10 days) while LOS increased to 11 days in those patients undergoing a surgical procedure (IQR 8 to 21 days, *p* = 0.008).

### Infection severity and classification

IDSA moderate infection was the most common presentation regardless of contact status (*n* = 105 of 156, 67 %). IDSA severe infection presentations occurred in 34 % (*n* = 39 of 116) of the no contact group and 30 % of the contact group (*n* = 12 of 40, *p* = 0.67), however no contact patients presenting with IDSA severe infection required greater numbers of lower extremity amputation with odds of 4.9 times higher than those with contact to a MDT-HRFS (no contact with severe infection and amputation = 34, 82.9 % vs. contact with severe infection and amputation = 7, 17.1 %, OR 4.9, 95 % CI 1.1 to 21.4, *p* = 0.037).

### Laboratory data

Inflammatory markers were similar between groups, with no significant difference; white cell count (no contact = 12.1 x *10^9/L*, IQR 10 to 15 vs. contact =10.7 x *10^9/L*, IQR 8 to 15, *p* = 0.241, 95%CI-0.87 to 3.45), erythrocyte sedimentation rate (no contact = 57 mm/min, IQR 26 to 77 vs. contact = 57 mm/min, IQR 34 to 86, *p* = 0.69), C-reactive protein (no contact = 76 mg/L, IQR 32 to 172 vs. contact = 70 mg/L, IQR 23 to 155, *p* = 0.99).

### Culture results

Two hundred fifteen pathogens of suspected infection were isolated from soft tissue and bone cultures in 129 patients. Polymicrobial infections were more common than monomicrobial infections (monomicrobial = 44 patients, 28.2 % versus polymicrobial = 85 patients, 54.5 %). Culture results were unavailable for 27 patients (17.3 %) who all received empiric antimicrobial therapy.

Gram-positive cocci were the predominating pathogens with the combination of *Methicillin-sensitive Staphylococcus aureus* (MSSA) and *Methicillin-resistant Staphylococcus aureus* (MRSA) accounting for half of all gram-positive cultures (MSSA = 37 of 118, 31 % and MRSA = 31 of 118, 26 %). Gram-negative bacteria represented 47 % of cultures with gram-negative rods predominating as probable colonisers (71 %, 69 of 97).

### Cost analysis

The estimated total cost of foot infection in this cohort was $4,264,214. The total cost was significantly higher in the no contact $ 3,169,083 primarily due to the higher rate of patient presentation and surgical intervention. In comparison, the contact group costs amounted to 97 % less in total costs, ($1,095,131) however, the average cost per patient separation was similar between groups (no contact = $ 22,475 vs. contact = $21,473, *p* = 0.763).

## Discussion

While evidence exists for reduction of diabetes related lower extremity amputation through the use of multidisciplinary high risk foot care services [10, 23, 24] the link between reduction in infections and the use of multidisciplinary foot services is less clear. This retrospective study suggests access to the MDT-HRFS within the hospital at the time of admission for DFI provides similar clinical outcomes regardless of contact status prior to admission. However, in this study patients with DFI who had no previous contact with a MDT-HRFS constituted around three times the number of hospital admissions for lower extremity amputation compared to those with prior contact. Whilst this rate of lower extremity amputation was not significantly different between the two groups, population estimates suggest that the number of expected amputations in the no contact group should have been 55, however the observed number was 77, 22 more than expected.

The large numbers of patients with no contact to MDT-HRFS within a large district in Sydney, Australia is of concern, particularly with four services in our District, with a no-wait policy for urgent referrals. Population estimates suggest this group are over represented in relation to admission and amputation rates. Screening is the key to identify people with diabetes who are at risk of ulceration and complication [25]. This has been emphasised in many health policy documents [7, 8]. A large component of this work is often undertaken within the primary care setting. Whilst the baseline characteristics of the contact and no contact group were similar, it is surprising given the long standing duration of diabetes and co-morbidities that referral to a MDT-HRFS did not routinely occur.

Our results are consistent with the contention that MDT-HRFS reduce the risk of amputation particularly in association with severe infection. Severe DFI was associated with a five-fold increase in the odds of requiring a lower extremity amputation. Using the same criteria for grading severity of infection as in this study, Wukich and colleagues evaluated the outcomes of patients with moderate and severe DFI. Their retrospective study of 119 patients reported a similar seven-fold increase in the risk of patients undergoing amputation if they presented with severe DFI (7.12 RR 95 % CI 1.83-41.05) [26].

The median LOS in patients with no contact was three days longer than those with contact. While this was not significant between the groups the additional hospital days are associated with an increased burden on the healthcare system and this number is higher than national averages for admission with cellulitis without complications [27]. The most likely explanation for this finding is the higher number of surgical procedures undertaken during admission in this study population.

This study has limitations that should be noted. The retrospective design relies heavily on both the ability of the treating team and the clinical coding team to accurately capture all relevant patient data and assign a correct primary diagnosis [26]. Errors may also occur in the ICD-10 conversion to the correct DRG [28]. A prospective study would allow greater accuracy in coding and classification.

The AR-DRG clinical coding which is used to estimate healthcare costs can be influenced by under and over-coding and by coding errors. In this study, these problems were avoided by allocating AR-DRG codes for each submission after manually cross-referencing all of the data between the ICD-10 search, eMR and patient paper charts, rather than relying solely on the patient paper charts.

## Conclusion

This single-centre study indicates that patients with no contact with a multi-disciplinary high risk foot service account for around 75 % of hospital admissions and amputations for DFI. Given the evidence about the effectiveness of MDT-HRFS and the need for early identification and prevention the over representation of the no contact group is of concern. Appropriate referral and early access to these specialist clinics is needed. Primary health carers and general practitioners should be aware of patients that should be referred to MDT-HRFS. Future studies should be prospective and a multi-site study would provide a national perspective. Studies should also explore the potential barriers to early referral for those at risk of admission for complications of diabetes foot disease.
